# Comparing the Feasibility and Acceptability of a Virtual Human, Teletherapy, and an e-Manual in Delivering a Stress Management Intervention to Distressed Adult Women: Pilot Study

**DOI:** 10.2196/42390

**Published:** 2023-02-09

**Authors:** Kate Loveys, Michael Antoni, Liesje Donkin, Mark Sagar, Elizabeth Broadbent

**Affiliations:** 1 Department of Psychological Medicine The University of Auckland Auckland New Zealand; 2 Soul Machines Ltd Auckland New Zealand; 3 Center for Psycho-Oncology Research The University of Miami Coral Gables, FL United States; 4 Auckland Bioengineering Institute The University of Auckland Auckland New Zealand

**Keywords:** stress, cognitive behavioral stress management, telehealth, conversational agent, virtual human, e-manual, pilot randomized controlled trial

## Abstract

**Background:**

Virtual humans (VHs), teletherapy, and self-guided e-manuals may increase the accessibility of psychological interventions. However, there is limited research on how these technologies compare in terms of their feasibility and acceptability in delivering stress management interventions.

**Objective:**

We conducted a preliminary comparison of the feasibility and acceptability of a VH, teletherapy, and an e-manual at delivering 1 module of cognitive behavioral stress management (CBSM) to evaluate the feasibility of the trial methodology in preparation for a future randomized controlled trial (RCT).

**Methods:**

A pilot RCT was conducted with a parallel, mixed design. A community sample of distressed adult women were randomly allocated to receive 1 session of CBSM involving training in cognitive and behavioral techniques by a VH, teletherapy, or an e-manual plus homework over 2 weeks. Data were collected on the feasibility of the intervention technologies (technical support and homework access), trial methods (recruitment methods, questionnaire completion, and methodological difficulty observations), intervention acceptability (intervention completion, self-report ratings, therapist rapport, and trust), and acceptability of the trial methods (self-report ratings and observations). Qualitative data in the form of written responses to open-ended questions were collected to enrich and clarify the findings on intervention acceptability.

**Results:**

Overall, 38 participants’ data were analyzed. A VH (n=12), teletherapy (n=12), and an e-manual (n=14) were found to be feasible and acceptable for delivering 1 session of CBSM to distressed adult women based on the overall quantitative and qualitative findings. Technical difficulties were minimal and did not affect intervention completion, and no significant differences were found between the conditions (*P*=.31). The methodology was feasible, although improvements were identified for a future trial. All conditions achieved good satisfaction and perceived engagement ratings, and no significant group differences were found (*P*>.40). Participants had similar willingness to recommend each technology (*P*=.64). There was a nonsignificant trend toward participants feeling more open to using the VH and e-manual from home than teletherapy (*P*=.10). Rapport (*P*<.001) and trust (*P*=.048) were greater with the human teletherapist than with the VH. The qualitative findings enriched the quantitative results by revealing the unique strengths and limitations of each technology that may have influenced acceptability.

**Conclusions:**

A VH, teletherapy, and a self-guided e-manual were found to be feasible and acceptable methods of delivering 1 session of a stress management intervention to a community sample of adult women. The technologies were found to have unique strengths and limitations that may affect which works best for whom and in what circumstances. Future research should test additional CBSM modules for delivery by these technologies and conduct a larger RCT to compare their feasibility, acceptability, and effectiveness when delivering a longer home-based stress management program.

**Trial Registration:**

Australian New Zealand Clinical Trials Registry ACTRN12620000859987; https://www.anzctr.org.au/Trial/Registration/TrialReview.aspx?id=380114&isReview=true

## Introduction

### Background

Virtual humans (VHs) are sophisticated conversational agents with a realistic and humanlike animated appearance and behavior, and multimodal sensing of user states based on real-time interaction data (eg, speech prosody, facial expressions, language, and physiological data), which may increase engagement [1]. VHs are machine-controlled, autonomous animations typically presented on a computer, tablet, or smartphone screen; however, they can be presented in alternative delivery environments, such as augmented or virtual reality. VHs incorporate artificial intelligence techniques to inform their social interactions. For example, predicting a negative emotional state in a user may create an empathetic facial expression in the VH. They can respond to user data with multimodal communication cues (eg, facial expressions, speech, and gestures) to show empathy and understanding and build rapport. A VH differs from other similar interactive technologies, such as chatbots (text- or voice-only dialogue agents [2]) and avatars (animated agents that represent users in virtual environments [3]).

VHs are starting to be tested as a method of delivering health interventions because of their scalability and their social and emotional skills to encourage engagement. A review of preliminary studies suggests that eHealth interventions with VHs can be more effective than eHealth interventions without VHs for a variety of health outcomes with small effect sizes [4]. These are predominantly used not only in applications for behavioral medicine and health psychology but also in providing decision support and improving health literacy. However, the research field is developmental, and more studies are needed.

A VH may have advantages over other common technologies for delivering remote health interventions, such as telehealth and e-manuals. An intervention that is delivered by a VH may be more engaging than a self-guided e-manual yet more scalable and less expensive than video calling with a human therapist. VHs could be as engaging as human therapists present via webcams, given their ability to deliver humanlike social cues and respond to incoming social data. However, research is needed to understand whether VHs are a feasible and acceptable way to deliver complex psychological interventions. It is possible that issues pertaining to the uncanny valley or other usability challenges could arise [5], which are important to identify early in the development process with potential users as part of a co-design process. Moreover, studies have yet to evaluate how VHs compare with telehealth and self-guided e-manuals.

### Use Case: Cognitive Behavioral Stress Management

One application for which VHs may be useful is in the delivery of cognitive behavioral stress management (CBSM). CBSM is a stress management program that has been shown to be effective in a variety of populations, including women with breast cancer [6], people living with HIV or AIDS [7], men with prostate cancer [8], and individuals with chronic fatigue syndrome [9]. CBSM involves cognitive and behavioral exercises and interpersonal skills training and has been associated with significant improvements in distress, stress management skills, mood, and quality of life, as well as neuroendocrine, inflammatory, and immune function in these populations [7,10-12]. It has been shown to be an effective intervention with in-person delivery; however, the accessibility of in-person care can be limited by barriers such as social restrictions from a pandemic, cost, stigma, and living rurally [13-15].

Technology could increase the reach of CBSM, especially when access to in-person care is limited. Emerging evidence suggests that CBSM delivered through technology and human facilitation may improve psychological and physiological health outcomes [16-23]. CBSM delivered by a web-based collaborative care program has been found to improve depression, pain, quality of life, and immune markers, including interleukin-6, interleukin-1β, and natural killer cell numbers in patients with advanced cancer [19]. The collaborative care program involved an internet-based intervention supplemented by low-frequency in-person sessions with a therapist. Another study found that CBSM delivered by videoconferencing with a therapist improved stress to a moderate degree [24]. Similarly, CBSM delivered by a therapist over the telephone reduced stress in patients with chronic fatigue syndrome [18]. CBSM delivered by a therapist over an e-tablet app demonstrated feasibility, acceptability, and preliminary efficacy in a group of older women undergoing breast cancer treatment [20].

There is limited research on CBSM delivery using technology without therapist facilitation. However, 1 stress management intervention delivered through a one-on-one face-to-face orientation session, 10 app-based CBSM modules, and 2 follow-up phone calls showed preliminary efficacy in reducing stress and improving quality of life in cancer survivors, though it did not affect depression or anxiety [25]. There is more research evaluating self-guided, technology-based delivery of other psychological therapies, and the results suggest that this may be an appropriate way to deliver stress management interventions to a range of populations [26]. However, this research field is developmental, and more high-quality trials are needed. Moreover, computerized forms of therapy often have lower engagement and adherence rates outside of a clinical trial context [27,28]. This could be owing to factors such as a lack of social accountability or the engagement that a therapist would usually provide. VHs have yet to be evaluated for delivering CBSM; however, promising results have been found for other psychological interventions [4]. It is unclear how VHs would compare with telehealth and self-guided e-manuals in delivering a stress management intervention. To date, most of the literature consists of trials without an active comparator or with a pre-post–only design [4]. Trials are needed to compare VHs with active comparator conditions to show whether people find them equally feasible and acceptable.

### Study Aims

This study aimed to compare the feasibility and acceptability of a virtual human cognitive behavioral stress management (VH-CBSM), teletherapy (ie, a human therapist over video call; teletherapy cognitive behavioral stress management [T-CBSM]), and a self-guided e-manual (e-manual cognitive behavioral stress management [E-CBSM]) in delivering 1 session of CBSM to a community sample of adult women experiencing distress to gather feedback for ongoing development. We focused on adult women as they have been shown to have higher rates of distress than men in the general population [29], including during the COVID-19 pandemic when this study was conducted [30]. It was hypothesized that all 3 technologies would be feasible and acceptable delivery methods for 1 session of a stress management program. This study also investigated the feasibility and acceptability of the methodology in preparation for a future randomized controlled trial (RCT) of a full CBSM program.

## Methods

This study was conducted in accordance with the CONSORT (Consolidated Standards of Reporting Trials) 2010 statement extension for randomized pilot and feasibility trials [31] and the Journal Article Reporting Standards for Mixed Methods Research [32].

### Ethics Approval

Ethics approval was obtained from the University of Auckland Human Participants Ethics Committee on December 17, 2019 (reference no. 024085). The Australia New Zealand Clinical Trials Registry provided prospective registration of the trial on August 28, 2020 (registration no. ACTRN12620000859987). The participants provided written informed consent through a web-based form hosted on a secure survey website (Qualtrics). Potential participants were provided the opportunity to ask questions about the research before they consented. All data were deidentified with identification codes and stored separately to consent forms. The data remain confidential to the researchers. The participants were gifted with an NZ $30 (US $19) shopping voucher.

### Study Design

A pilot RCT was conducted with a parallel, mixed design involving 3 conditions (VH-CBSM, T-CBSM, and E-CBSM). As a mixed methods study, a convergent, complementary design was adopted, in which the qualitative data served to enrich and explain the quantitative findings [33,34]. The primary outcomes of this study were feasibility and acceptability. Data were also collected on psychological and physiological stress, distress, optimism, and stress management skills, and the results are presented elsewhere [35]. There were no significant changes in the trial methods after commencement.

### Participants

In total, 43 participants were recruited. The inclusion criteria were females aged ≥30 years with English fluency who self-identified as feeling stressed (based on their own perception). Women aged ≥30 years were recruited, as the intervention was ultimately intended to be used by middle-aged women. Participants were recruited through a university staff email list and research website and by word of mouth. The eligibility screening took place via email.

A recruitment target of 36 participants was set (12 participants per group) as recommended for pilot trials [36]. Julious [36] proposed a popular method that suits instances where there is limited previous information with which to base a sample size calculation. In total, 43 participants (ie, 7 additional participants) were recruited for the following reasons: (1) to account for 2 participants who lost contact with the study before their appointment, (2) to account for 1 participant who was an outlier, and (3) to account for 4 participants whose physiological data were not collected owing to a sensor error. Recruitment was stopped once the quota of 36 participants with complete data was met. Recruitment was conducted between April 12 and May 14, 2021, and data were collected between April 13 and May 28, 2021.

### Randomization

Participants were randomly allocated to 1 of the 3 conditions using a 1:1:1 allocation ratio with Research Randomizer software, which was performed by a researcher not involved in data collection (EB). The allocations were concealed in sealed opaque envelopes from the researcher who enrolled participants (KL), until after participants had enrolled and before scheduling their in-person appointment. A single-blind study was necessary for the researcher (KL) to coordinate booking times with the human teletherapist where applicable. However, data collection was conducted in a manner that minimized bias (eg, the researcher followed a script during the appointment, and data from the appointment were collected using deidentified web-based forms that participants completed independently with the screen out of the researcher’s view). The participants were blinded to their condition until the start of the intervention.

### Procedure

The participants attended a 1- to 2-hour appointment in a private room at the University of Auckland Clinical Research Centre. The session was hosted in a research center to enable tight control over the setting in which the technologies were receiving a preliminary feasibility evaluation without extraneous variables affecting the results (eg, internet speed, computer processing power, and distractions). This also enabled the researcher to quickly record and assist with any errors from the prototype technologies. Multimedia Appendix 1 depicts the procedure from the baseline assessment to the 2-week follow-up assessment, including details of what occurred during the appointment and 2-week follow-up period.

The participants received identical content across the conditions, which included 1 session of CBSM. The session combined selected content from modules 1 and 2 of the “V-SMART Video-Conferenced Stress Management and Relaxation Training” manual [20,37]. The session provided information and cognitive exercises to improve stress awareness, psychoeducation about the benefits of deep breathing, and a deep breathing exercise. All participants received a printed manual containing summary information, stress awareness checklists and exercises, and a guide to plan their at-home deep breathing practice. The intervention content, including language, was identical across conditions; all that varied was the delivery format. This served the purpose of providing a controlled comparison between the delivery methods without the addition of extraneous variables affecting the results (eg, deviations from the intervention content or additional rapport building from small talk in the teletherapy condition).

During the 2-week follow-up period, participants were provided with a daily stress measure and homework to complete (Multimedia Appendix 1). The participants were emailed a link to a password-protected homework website that they were asked to visit over the following 2 weeks. The website contained 2 educational videos about stress awareness and 1 video of a deep breathing exercise that was approximately 8 minutes in length. The videos were produced by the University of Miami as part of a previous CBSM trial [20]. Participants were asked to practice the deep breathing exercise daily.

### Intervention Conditions

#### Virtual Human

Sam, the VH facilitator, was developed by Soul Machines Ltd. Soul Machines VH are autonomous human animations modeled on the features of real humans using light room technology and computer-generated imagery techniques [38]. They contain an elaborate cognitive architecture modeled on the human brain driven by artificial neural networks [39]; continuously analyze user data during an interaction to inform their social and emotional responses; classify users’ emotional states based on their speech, language, facial expressions, and gestures using live neural networks; and autonomously respond with speech, facial behaviors, and gestures that are emotionally appropriate to the interaction. This enables them to display communication cues consistent with empathy and understanding, which may help build rapport, acceptability, and engagement with the content that they deliver.

**Figure 1:**
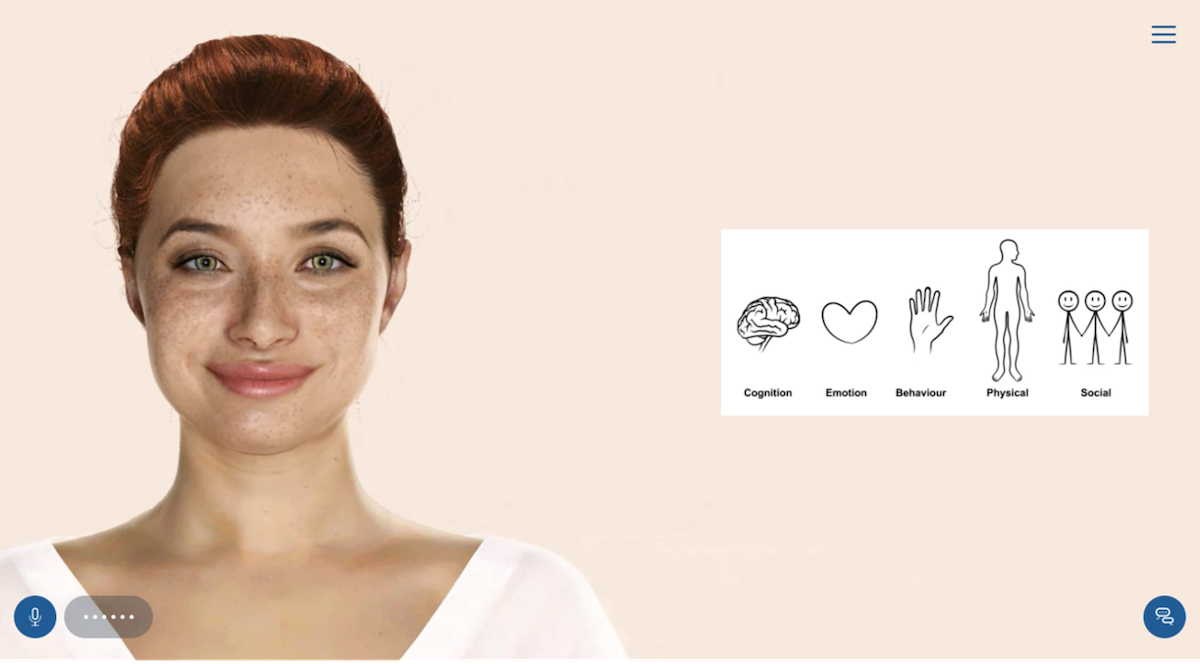
The Digital Human interface (“Sam”) while presenting a diagram on the 5 ways that stress manifests in the body.

The VH in this study, Sam, was modeled as a young adult, White female and synthesized from the physical features of multiple human models (ie, she was not modeled on a particular person; Figure 1). She was presented on an internet browser.

Sam interacted autonomously with participants using a finite-state conversation engine with preprogrammed responses. This conversation design enabled greater experimental control, as Sam followed an intervention script. Participants were provided with relatively fixed multiple-choice response options throughout the interaction, and Sam tailored her responses to the participants’ answers. Participants could interact with Sam using speech, typing, or clicking response options on the screen. Sam would respond in speech, although a text copy of her speech could be viewed if participants opened the messenger window on her interface. Sam spoke using a computer-generated voice with an Australian accent (“Microsoft Natasha—female [neural]”). The voice sounded relatively humanlike, as it used neural networks to inform speech intonation. If Sam did not understand a participant’s response, she would say a variation of, “I’m sorry, I didn’t understand. Could you repeat or reword your statement?” After 2 failed attempts, she would ask participants if they would like to move to a different part of the conversation and provided them with a menu of areas that they could move to (eg, stress video or deep breathing exercise).

Sam delivered emotional expressions on her face, including compassion, joy, and concern. Her emotional responses were preprogrammed using a text-to-speech Emotion Markup Language for consistency between participants [40]. The Emotion Markup Language triggered emotional expressions in Sam’s face from her speech content. Sam engaged in humanlike movements such as holding eye gaze, eyebrow raises, head tilts, and shoulder movements. Sam’s facial expressions and body movements were autonomously generated in real time using a visual computing framework and neurobehavioral modeling techniques [39,41]. Sam engaged in rapport-building strategies throughout the intervention, informed by Parks and Floyd [42] and Loveys et al [43], including emotional expressiveness, empathy, compassion (in language and facial expression), being nonjudgmental, and providing praise and support.

Sam presented illustrations to demonstrate concepts (eg, the 5 ways that stress manifests; Figure 1) and a 20-minute video on how stress and stress management techniques affect the body. Sam also delivered an audio recording of a deep breathing exercise, which was approximately 7 minutes in length. The deep breathing exercise was recorded by a young female master’s level trainee health psychologist (different from the human teletherapist).

Sam continuously collected audiovisual data to hear participants’ speech and see facial expressions. These data were classified by the system in real time to inform its immediate responses, but the data were not stored. The system’s data processes were in accordance with the European Union’s General Data Protection Regulation [44,45].

#### Teletherapy

In the teletherapy condition, the intervention was delivered over Zoom videoconferencing software by a teletherapist, who was a master’s level trainee health psychologist, and a young adult, White female. The teletherapist was called from a private room in front of a white background. The participants were unaware of the location of the teletherapist. She referred to an on-screen script to ensure that the intervention content was consistent across conditions and participants. This approach has been taken in previous telehealth trials [46] and serves to reduce confounding factors between conditions (eg, deviations from planned therapy content). During the 10-minute break in the middle of the session, the therapist muted her microphone and turned off her camera to prevent chit chat. The therapist was trained and supervised by a senior clinical health psychologist. She was trained to respond to severe distress or suicidal ideation across conditions; however, none of the participants reported experiencing this.

#### e-Manual

**Figure 2:**
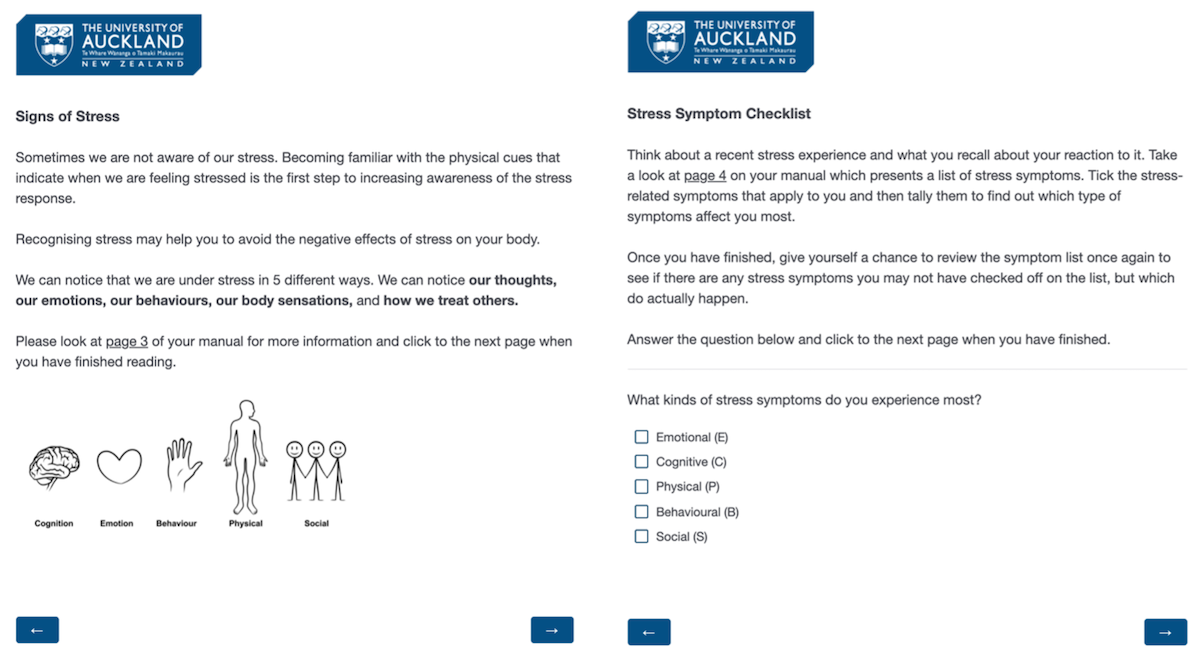
Examples of the e-Manual interface.

The self-guided e-manual was hosted on Qualtrics (Figure 2). Information was delivered in text alongside illustrations with click-through pages, check boxes to answer questions, and a link to a deep breathing audio recording. The e-manual delivered information identical to that of the other conditions.

### Measures

#### Overview

All self-reported data were collected electronically on a secure survey website (Qualtrics). The participants completed the baseline, daily stress, and 2-week follow-up assessments at home. The postintervention assessment was administered during the in-person appointment. Overall, this study evaluated the feasibility of the intervention delivery methods and trial methods, where feasibility is defined as the extent to which an intervention or trial methodology can be practically implemented [47]. This study also examined the acceptability of the intervention and aspects of the trial methodology. Acceptability is a multidimensional construct referring to the extent to which users perceive a health intervention or trial methodology as appropriate [48].

Our criteria for determining the feasibility of the intervention were based on quantitative data and included the following: (1) ≤30% of participants using the technology encountered a technical error and (2) ≥80% of participants could access the homework. To determine the feasibility of the trial methods, we required the following: (1) our recruitment target of 36 participants to be met and (2) for no more than 30% attrition. Our acceptability criteria were as follows: (1) mean scores should be above the midpoint on self-report measures, where higher scores indicate greater acceptability, and (2) intervention completion should be above 80%.

#### Feasibility: Intervention

The number of times the researcher provided technical support during the session was counted. The feasibility of the homework was evaluated using 2 questions (“Were you able to access the study website from home?” “Were you able to access the homework videos from home?”) with a yes or no response option at follow-up.

#### Feasibility: Trial Methods

The number of participants recruited from each method, missing data, and issues with questionnaire completion were assessed along with observations of methodological difficulties experienced during the study.

#### Acceptability: Intervention

Intervention acceptability was assessed using self-report and behavioral measures. For the self-report measures, 2 items evaluated satisfaction with the resource for improving stress management and the likelihood of using the resource’s suggestions in the future to help cope with stress [49]. Responses ranged from 1 (“not satisfied” or “definitely no”) to 7 (“very satisfied” or “definitely yes”). Four 100-mm Visual Analog Scales (VASs) were used to evaluate willingness to participate in a 10-week and 5-week program, comfort with performing the intervention alone at home, and willingness to complete the intervention in a group setting from 0 (“not at all”) to 100 (“definitely”). Participants rated how engaging they found the intervention on a 100-mm VAS from 0 (“not at all”) to 100 (“extremely”). Participants were asked about their willingness to recommend the intervention to someone that they care about in an open-ended question, and responses were coded into “yes,” “no,” or “in certain circumstances” based on the content of each participants’ language. Behavioral measures of acceptability included a count of the number of participants who completed the full session and self-reported homework completion (“all” or “some” or “none” response options) and whether each homework video was watched (“yes” or “no” response options).

#### Acceptability: Therapist Relationship

Rapport and trust with the VH and human teletherapists were assessed using the Working Alliance Inventory bond subscale (Working Alliance Inventory [WAI] [50]). The WAI bond subscale has been shown to have good internal consistency reliability (α=.85-.92) and concurrent validity, and it has been used previously with conversational agents [51]. It consists of 12 items scored from 1 (“never”) to 7 (“always”). The total scores can range from 12 to 84, where a higher score indicates greater rapport [52]. The scores were interpreted as low (12-36), moderate (37-60), or high (61-84). Trust was measured using a 100-mm VAS, “I trusted my therapist” from 0 (“not at all”) to 100 (“extremely”).

#### Acceptability: Trial Methods

Participants reported how comfortable they felt with being audio-recorded during their appointment for the purpose of informing improvements to the intervention software. Participants responded using a 100-mm VAS, from 0 (“Not at all”) to 100 (“Extremely”), and were provided with space to elaborate on their responses in writing. Moreover, the researcher made observations on the acceptability of the methodology during the trial based on feedback communicated by the participants.

#### Acceptability: Qualitative Data

Qualitative data were collected to enrich and clarify the quantitative data on acceptability outcomes. The postintervention questionnaire included 3 open-ended questions that evaluated the following: (1) what participants’ favorite activity was in the intervention session, (2) what participants liked about the delivery of their intervention session, and (3) how the delivery of the intervention could be improved. Within the 2-week follow-up questionnaire, 2 open-ended questions evaluated which aspects of the homework exercises the participants liked and how they thought the homework could be improved.

### Data Analysis

#### Quantitative Data

Data were analyzed using IBM SPSS software (version 27). Missing data were handled by mean imputation according to Little's Missing Completely at Random (MCAR) test, and the data were missing completely at random (*P*>.99). For instances where mean imputation was not possible (eg, 1-item measures asking whether a homework video was watched), the data were not included in the analysis. Data were checked for violations of test assumptions, and the assumptions were met. Baseline sample characteristics were calculated for the overall sample and compared between groups using chi-square tests and 1-way ANOVA tests. Intervention acceptability, working alliance, and therapist trust were compared between groups using 1-way ANOVA tests. Post hoc tests with Bonferroni correction were used to investigate significant effects. Feasibility and behavioral engagement were compared between groups using chi-square tests.

#### Qualitative Data

The Braun and Clarke [53] approach to reflexive thematic analysis was used to analyze written responses to 4 open-ended questions. The steps were completed by 1 researcher (KL) as per recommendations. An inductive approach was used to generate themes, informed by data content. Themes were generated separately by condition for questions about the delivery methods, and for the whole sample for questions about the intervention content and homework. Themes were reviewed to ensure internal coherence and distinction from other themes and combined or split to improve specificity where necessary.

#### Mixed Methods Data Integration

Integration of quantitative and qualitative data occurred through the research design, interpretation, and reporting procedures. In terms of research design, data were connected through the sampling frame (ie, the data were collected concurrently from the same participants) [54]. Data were then analyzed and reported using a contiguous approach [54]. The coherence of the quantitative and qualitative findings was evaluated using a narrative format.

## Results

### Participants

**Figure 3:**
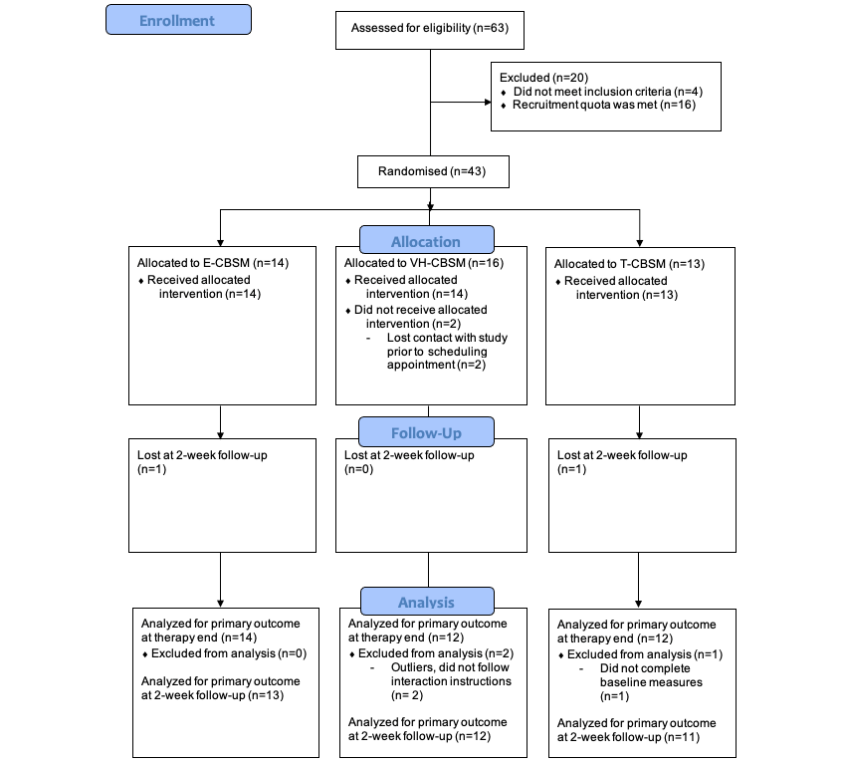
CONSORT (Consolidated Standards of Reporting Trials) diagram of participant flow.

Table 1 presents the demographic and psychological characteristics of the participants. The participants were 38 females of predominantly New Zealand European or Asian ethnicity. Most participants worked full-time and were highly educated. Most were married or living with a partner. In total, 11 participants reported a mental health diagnosis (4=major depression; 5=generalized anxiety; and 2=depression and anxiety). There were no significant group differences in baseline demographics or psychological variables (all *P*>.119). A CONSORT diagram depicts the participant flow (Figure 3). All participants lived in a private dwelling with internet access. Only 16% (6/38) of participants lived alone. All participants had access to a computer at home with a microphone, and 95% (36/38) had a webcam.

**Table 1:** Participant characteristics (N=38).

Characteristics			Total		Condition				
					e-Manual (n=14)		Virtual human (n=12)		Teletherapy (n=12)
Age (years), mean (SD)			43.21 (10.70)		44.71 (11.93)		39.67 (7.97)		45.00 (11.55)
**Ethnicity, n (%)**									
	New Zealand European	24 (63)		8 (57)		8 (67)		8 (67)	
	Asian	9 (24)		5 (36)		2 (17)		2 (17)	
	Māori	3 (8)		1 (7)		2 (17)		0 (0)	
	Middle Eastern, Latin American, or African	1 (3)		0 (0)		0 (0)		1 (8)	
	Other	1 (3)		0 (0)		0 (0)		1 (8)	
**Education level, n (%)**									
	High school or less	1 (3)		1 (7)		0 (0)		0 (0)	
	Trade qualification	2 (5)		1 (7)		1 (8)		0 (0)	
	Undergraduate degree	12 (32)		6 (43)		3 (25)		3 (25)	
	Postgraduate degree	23 (61)		6 (43)		8 (67)		9 (75)	
**Marital status, n (%)**									
	Single	8 (21)		6 (43)		1 (8)		1 (8)	
	Relationship	5 (13)		1 (7)		3 (25)		1 (8)	
	Married or living with partner	21 (55)		5 (36)		7 (58)		9 (75)	
	Separated or divorced	4 (11)		2 (14)		1 (8)		1 (8)	
**Work status, n (%)**									
	Full-time	27 (71)		10 (71)		9 (75)		8 (67)	
	Part-time	6 (16)		3 (21)		2 (17)		1 (8)	
	Beneficiary	1 (3)		1 (7)		0 (0)		0 (0)	
	Unemployed	4 (11)		0 (0)		1 (8)		3 (25)	
**Mental health, n (%)**									
	At least 1 mental health diagnosis	11 (29)		4 (29)		3 (25)		4 (33)	

### Feasibility: Intervention (Technical Issues)

The delivery method did not significantly affect whether technical support was sought (*χ^2^*_2_=2.37, *P*=.31). Technical support was sought 4 times for the e-manual (navigation issues) and 4 times for the VH (frozen or jumped conversations). In the teletherapy condition, 1 video lag issue was reported, although technical support was not sought. The technical issues were relatively minor and brief and did not affect intervention completion. Of the participants who completed the 2-week follow-up measure, most (32/36, 89%) reported that they were able to access the study website and homework videos from home.

### Feasibility: Trial Methods

#### Recruitment Methods

The recruitment target of 36 participants was met, as 43 participants were recruited. The most successful recruitment method was an advertisement to a university staff email list (37/43, 86%), followed by a research recruitment website (3/43, 7%), word of mouth (2/43, 5%), and a Facebook advertisement (1/43, 2%).

#### Measurement Tools

Only 1 participant who was scheduled for an appointment did not complete the baseline questionnaire. The response rates were high for the 2-week follow-up assessment (36 of 38 respondents, 95%). However, 3 participants did not complete most of the follow-up questionnaires. Only 18 of the 38 (47%) participants responded to all 13 days of daily stress measure. The researcher observed that several participants reported issues completing the WAI bond subscale during the in-person appointment, both in relation to a VH and after only 1 interaction with a human teletherapist. Five participants wanted a “not applicable” option added: 3 for the VH and 2 for the human teletherapist conditions. Therefore, the validity and reliability of the scale may have been impaired. It is possible that this measure is best suited to long-term interactions, and its validity in VH should be further examined.

#### Observations of Methodological Difficulties

During the appointment, the researcher observed that 1 participant experienced difficulty in using a laptop, including the scrolling function. After this, the researcher verified that each participant was familiar with the laptop design and demonstrated how to use it. Although being personal preferences, 2 participants reported that they would have preferred to use a desktop computer with a larger screen and an ergonomic keyboard.

### Acceptability: Intervention

The means, SEs, and CIs for the acceptability results are reported in Table 2. There were no significant differences in satisfaction between the groups (*F*_2,35_=0.95, *P*=.40, η_p_^2^=0.05). All conditions received good satisfaction ratings. Participants indicated that they would be very likely to use the intervention’s techniques to cope with stress in the future, with no significant differences between groups (*F*_2,35_=0.01, *P*=.99, η_p_^2^=0.00).

**Table 2:** Means, SEs, and 95% CI values for the acceptability results.

Variables		Total sample	Condition		
			E-CBSM^a^	VH-CBSM^b^	T-CBSM^c^
**Acceptability: intervention, mean (SE); 95% CI**					
	Satisfaction with resource	5.43 (0.22); 4.98-5.87	5.36 (0.36); 4.63-6.09	5.08 (0.39); 4.29-5.88	5.83 (0.39); 5.04-6.63
	Likelihood of using therapy techniques	6.18 (0.18); 5.82-6.54	6.21 (0.29); 5.62-6.81	6.17 (0.32); 5.53-6.81	6.17 (0.32); 5.53-6.81
	Willingness to complete a 5-week intervention	76.92 (4.60); 67.58-86.26	77.85 (7.75); 62.10-93.59	79.83 (8.06); 63.45-96.22	73.08 (8.06); 56.70-89.47
	Willingness to complete a 10-week intervention	69.34 (4.51); 60.19-78.49	65.93 (7.41); 50.89-80.97	72.50 (8.00); 56.26-88.74	69.58 (8.00); 53.34-85.83
	Comfort to complete at home	87.16 (3.03); 81.01-93.31	94.07 (4.98); 83.96-104.18	89.42 (5.38); 78.50-100.34	78.00 (5.38); 67.08-88.92
	Willingness to complete in a group setting	49.79 (6.20); 37.17-62.41	56.00 (10.30); 35.04-76.96	50.46 (11.20); 27.67-73.24	42.92 (10.72); 21.10-64.73
	Perceived engagement	72.91 (3.78); 65.24-80.58	75.14 (6.21); 62.54-87.75	67.50 (6.71); 53.89-81.11	76.08 (6.71); 62.47-89.70
Therapist rapport, mean (SE); 95% CI		N/A^d^	N/A	44.42 (15.22); 34.75-54.09	69.54 (11.66); 62.49-76.58
Therapist trust, mean (SE); 95% CI		N/A	N/A	65.42 (28.37); 47.39-83.44	85.23 (18.40); 74.11-96.35
**Acceptability: trial methods, mean (SE); 95% CI**					
	Comfort with audio recording	87.98 (2.92); 82.07-93.90	95.29 (4.79); 85.56-105.01	87.83 (5.17); 77.33-98.34	80.83 (5.17); 70.33-91.34

^a^E-CBSM: e-manual cognitive behavioral stress management.

^b^VH-CBSM: virtual human cognitive behavioral stress management.

^c^T-CBSM: teletherapy cognitive behavioral stress management.

^d^N/A: not applicable.

Overall, participants were significantly more willing to engage in a 5-week program than a 10-week program (*t*_36_=−3.23, *P*=.003). There was no significant effect of delivery method on participants’ willingness to participate in a 5-week (*F*_2,34_=.19, *P*=.83, η_p_^2^=0.01) or a 10-week version of the intervention (*F*_2,35_=.18, *P*=.83, η_p_^2^=0.01).

Overall, participants reported that they would be very comfortable completing the intervention alone from home (Table 2). There was a trend toward a difference between the groups, with a moderate-to-large effect size (*F*_2,35_=2.50, *P*=.10, η_p_^2^=0.13), however this was not statistically significant. Participants would be most comfortable using the e-manual and then the VH from home. Participants were the least comfortable with video calling a human therapist from home. Participants were ambivalent about completing the intervention in a group setting, with no significant effect of the delivery method on preferences (*F*_2,33_=.39, *P*=.68, η_p_^2^=0.02). There were no significant group differences in how engaging the participants perceived the intervention to be (*F*_2,35_=.50, *P*=.61, η_p_^2^=0.03). Overall, each delivery method was reasonably engaging.

**Figure 4:**
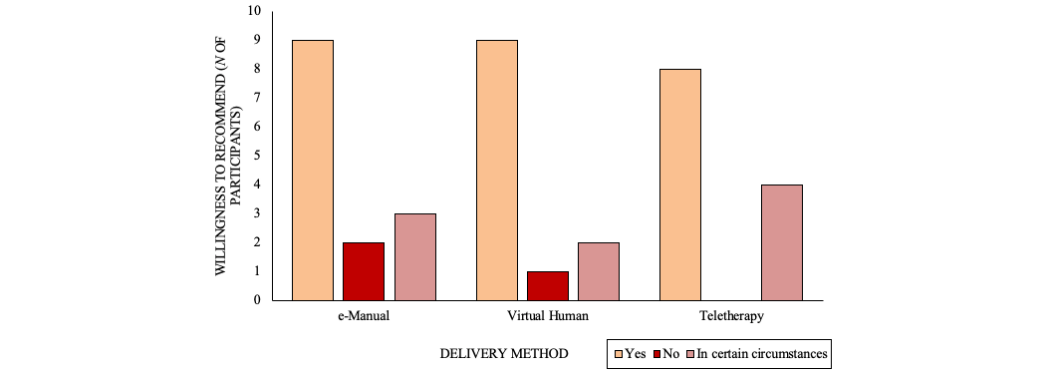
Willingness to recommend the intervention by delivery method.

Of the 38 participants, 26 (68%) reported that they would recommend the intervention to someone they cared about, 9 (24%) reported that they would recommend it in certain circumstances, and 3 (8%) reported that they would not. A chi-square test revealed no significant differences between the groups (N=38, *χ^2^*_4_=2.54, *P*=.64; Figure 4).

Regarding intervention completion, 41 participants attended the session, 39 (95%) of whom completed it fully. Two participants in the VH condition did not adhere to the instructions on how to interact with the VH nor to seek help if technical issues occurred. Therefore, the intervention was not fully delivered, and the deep breathing exercise was skipped for these 2 participants. Of the participants who responded, most were adherent to the homework exercises (16/32, 50% completed all; 13/32, 41% completed some; 3/32, 9% completed none). There was no significant group effect (N=32, *χ^2^*_4_=2.46, *P*=.65). Most participants watched homework video 1 (28/32, 88%), 2 (28/32, 88%) and 3 (26/32, 81%).

### Acceptability: Therapist Relationship

Therapist rapport was significantly greater with the human teletherapist than with VH (*t*_23_=−4.66, *P*≤.001, Cohen *d*=−1.87). The human teletherapist achieved high rapport, whereas the VH achieved moderate rapport. However, the results should be interpreted with caution as per the concerns pertaining to the evaluation measure discussed previously. Participants trusted the human teletherapist significantly more than the VH (*t*_23_=−2.09, *P*=.048, Cohen *d*=−0.84). However, both conditions received scores above the midpoint in rapport and trust variables.

### Acceptability: Trial Methods

#### Comfort With Audio Recording

Overall, participants reported feeling comfortable with being audio-recorded for informing improvements to the intervention software (Table 2). There were no significant group differences in the perceived acceptability of the audio recordings, although there was a moderate effect size (*F*_2,35_=2.11, *P*=.14, η_p_^2^=0.11). Participants in the e-manual condition reported feeling most comfortable with the audio recording compared with participants in the VH and teletherapy conditions. Participants generally accepted being audio-recorded if their privacy was protected. Most participants (n=26) reported that they did not mind being recorded, or forgot that they were being recorded (n=3). Only a few participants (n=4) reported feeling slightly more self-conscious. For example, Participant 017 reported, “It made me a little more self-aware but [it’s] not really a big deal.” Three participants reported that they would have been less comfortable if they had revealed more personal information.

#### Observations on Methodology

Three participants informed the researcher that they found the daily stress measure during the 2-week follow-up period was helpful for improving their stress awareness. One participant reported that she was continuing to monitor her daily stress on a 0 to 100 scale after the study and used this information to inform her self-care. Another participant reported that the daily stress measure helped her see the inaccuracy of her perception that she was consistently stressed.

### Acceptability: Qualitative Data

Most participants reported that their favorite activity was the deep breathing exercise (n=33). Other participants reported that their favorite activities were learning about the appraisal process (n=5), the effects of stress on the body (n=4), learning to identify stressful situations (n=3), reading the effects of stress (n=2), watching the educational video (n=2), completing the stress symptom checklist (n=1), and using the printed participant manual (n=1). Several participants reported having more than 1 activity as their favorite.

The participants identified several strengths and limitations of the intervention delivery methods and homework exercises. Definitions of the themes are presented in Multimedia Appendix 2. The themes, subthemes, and representative quotes are outlined in Multimedia Appendix 3. In summary, the strengths of teletherapy included access to a calm and attentive therapist, social connection, and ease of use (Table S3 in Multimedia Appendix 3). The VH was viewed as nonjudgmental, low in social pressure, and easy to use. The e-manual was valued for its privacy, self-determined pace, and low social pressure. In terms of the perceived limitations, all delivery methods could have been improved with simplified language, a shorter session, and more tailored suggestions (Table S4 in Multimedia Appendix 3). In the telehealth condition, participants requested an in-person session, whereas in the VH condition, participants would have preferred improved lip sync and slower speech speed during explanations. The e-manual delivery could have been improved by having a facilitator present, less reading, and more visual components. Regarding the homework, participants liked the simple breathing exercises, ease of use, and how the videos reinforced the content from the in-person session (Table S5 in Multimedia Appendix 3). Possible improvements included shorter and newer videos that did not repeat information, tailored to the local cultural context (Table S6 in Multimedia Appendix 3). Participants would also have liked to receive reminders to complete their homework.

### Data Integration

The findings from the quantitative and qualitative data confirm one another. The quantitative data revealed no significant difference between conditions on acceptability ratings (eg, satisfaction and willingness to use), with most ratings achieving a good score. This indicates that though the responses were generally positive toward the technologies, the participants perceived areas in which they could be improved. This is confirmed and explained in the qualitative data, in which participants identified many strengths and areas in which the delivery could be made more acceptable (eg, shorter sessions and simplified language). The high completion and low technical error rates recorded in the quantitative data were also confirmed in the qualitative results, in which participants reported that each technology was easy to use. Interestingly, participants did not report the technical errors that the researcher recorded during the session, suggesting that these were not viewed as salient issues by participants. Indeed, the researcher’s observations confirmed that the technical errors were minor.

## Discussion

### Principal Findings

Overall, this study found that a VH, teletherapy, and a self-guided e-manual were feasible and acceptable methods for delivering 1 brief session of CBSM to a community sample of distressed adult women, based on quantitative data enriched by insights from the qualitative data. There were no significant feasibility issues affecting the intervention or homework completion across conditions, and the feasibility criteria were met. Some minor technical issues were encountered in each condition that could be avoided in future studies through different hardware setups (eg, the use of ethernet cables for a stable internet connection). In a multisession trial, it is possible for participants to build familiarity with the technology and thus develop confidence in resolving any minor errors that could be encountered (eg, lag during a telehealth video call). Moreover, the capabilities of software, such as VH and telehealth, and the hardware through which they are delivered, are gradually improving with time, reducing the chance of errors in the future. Regarding the acceptability results, all conditions received high satisfaction, perceived engagement ratings, and completion rates. Most users were either willing or willing in certain circumstances to recommend the intervention to someone they cared about. There were no clear differences in the acceptability of the technologies for delivering a stress management session based on the quantitative data, and all conditions met the acceptability criteria.

The qualitative data added an extra dimension by showing that each technology had unique strengths and limitations that could have influenced its acceptability. According to the qualitative results, the VH was viewed as a nonjudgmental yet humanlike way of delivering the intervention in a manner that was easy to understand and use. However, a slower speech speed during explanations and more tailored responses would have enhanced acceptability. The teletherapist provided a calm, attentive, and comfortable interaction with a sense of human connection. However, participants would have liked the session to be in person and with more tailored advice. Regarding the self-guided e-manual, participants appreciated that it was a private, self-paced way to complete therapy without social pressure. However, they disliked the large amount of reading involved and would have preferred more visual components and a facilitator. On the basis of our quantitative and qualitative results, each technology was acceptable for delivering brief stress management support to adult women presenting with distress. The unique strengths and limitations of the technologies in a stress management application, as described in the qualitative data, could mean that the technology that is best suited to delivering a stress management application could vary depending on the preferences of the population, intervention techniques, and use contexts (eg, brief vs long-term support), and additional trials are needed to better understand the situations in which each of the technologies are best suited. Furthermore, the qualitative data revealed areas in which the intervention delivery could have been improved across conditions, including more simplified language, shorter sessions, and more tailored suggestions.

The results add to a growing body of literature showing the promise of VH, teletherapy, and self-guided e-manuals for delivering remote psychological therapies [4,37,55,56]. Conversational agents and self-guided internet interventions have been shown to be acceptable for delivering other types of psychological interventions in adults [57-59]. However, digital health interventions without therapist facilitation have been shown to have low adherence outside clinical trial contexts, which may be owing to the lack of social accountability [27]. Thus, long-term acceptance and engagement could be an issue for a self-guided e-manual.

VHs may be able to boost adherence to web-based interventions because of their social and relational capabilities. Indeed, a recent pilot trial observed high behavioral engagement in a VH intervention at home over a period of 1 week [60]. However, further studies are required to investigate this. On the basis of the qualitative data, participants did not experience an uncanny valley effect with the VH (ie, feelings of eeriness with noticing non-humanlike and abnormal features on humanlike artificial agents) [61], which may have helped acceptability. The accuracy of the VH in understanding user speech is a key contributor to its success in psychotherapeutic applications. Although we do not have data on the conversation error rate from this study, no participants sought technical support for misunderstanding nor was this mentioned as an issue in the qualitative data, suggesting that accuracy was acceptable. Sam was designed with a relatively closed-ended conversation with fixed response options, which may have reduced the chance of language understanding errors. However, future research could explore the effects of language understanding and conversation design on VH acceptability in stress management applications. The capabilities of VH are developing rapidly with advances in areas such as natural language processing and artificial intelligence techniques, computer processing power, and data availability. These advances enable new social capabilities that promote relationship building and engagement. The promising results highlight the potential of these highly scalable technologies to expand access to therapy.

The teletherapist received greater rapport and trust scores than the VH did. Therefore, additional research is needed to investigate which VH features could enhance rapport with users and, therefore, enhance acceptability and engagement. It remains an open question as to how conversational agents in health applications should be designed [62]. However, previous studies have suggested that a co-design process is required [63,64], and consideration of how user characteristics (eg, gender and ethnicity) and use context impact the perception of agent behaviors [43]. Moreover, specific agent features such as tailoring conversational agent characteristics to match user culture and personality [65,66], personalizing content [67], and expressing empathy [68] may boost rapport and engagement.

Although the teletherapy condition received promising results, there are inherent limitations of this technology in terms of increasing the accessibility of an intervention. For example, it may have reduced scalability and increased cost relative to other digital interventions [69]. Moreover, participants in this study reported feeling least comfortable completing a teletherapy session from home compared with the other technologies. This may be owing to concerns about privacy, outside interruptions (eg, from children, partners), or distractions from the computer or phone (eg, email notifications), as evidenced in several previous studies [70,71].

The trial methodology was largely found to be feasible; however, some issues were identified from the researcher observations and qualitative feedback. These issues pertained to the measurement of therapist rapport using the WAI bond subscale after 1 session, the absence of daily homework reminders, sending reminders to complete the daily stress measure over email, and having a time gap between watching the homework videos and their evaluation. Moreover, some participants requested a more comfortable environment in which to complete the intervention (eg, a more padded chair and a less clinical environment) and a desktop computer with a larger screen. In the real world, users of these technologies would be able to use their hardware of choice to access the intervention from home, therefore this would not be an issue for delivery. However, future trials might consider providing a desktop computer if collecting data from a research center or allowing people to use their preferred devices from home.

### Strengths and Limitations

A strength of this study was that it used a pilot RCT design with 3 active comparators. However, several limitations could affect the generalizability of the results. The sample population comprised highly educated women of predominantly New Zealand European or Asian ethnicities. It is unclear how well the results can be generalized to other genders and to the broader population. Moreover, it is unclear whether a clinical population would find the technology delivery methods acceptable or feasible, and additional trials are needed. Another limitation was that the technologies were evaluated after only 1 session. This approach was chosen to evaluate how the technologies and methodology worked before investing resources into developing additional therapy modules, which involves significant time and cost. It is possible that there could be greater variation in acceptability between the technologies with longer-term use. A self-guided e-manual may become less acceptable and engaging to people after 10 weeks of use compared with a VH or a teletherapist where a relationship is developed, and the experience is more dynamic. In addition, this study was conducted at a clinical research center to create a controlled environment for a preliminary feasibility evaluation without extraneous variables affecting the results and to enable the researcher to quickly monitor and assist with any technical difficulties with the prototypes. Future trials should evaluate the feasibility and acceptability of these technologies in more naturalistic environments (eg, users’ homes). Future trials might also compare the performance of a VH to an in-person therapist appointment to understand how effect sizes differ. Finally, the WAI bond subscale was used to evaluate therapist rapport because of its use in previous conversational agent studies. However, in this study, we found that this measure appeared to be less appropriate for evaluating one-off interactions. Although the WAI bond subscale would be suitable in longer trials involving several interactions with a teletherapist or VH, future studies involving 1 therapeutic interaction should use a different measure.

### Future Research

Our results indicate several areas for future research. Additional CBSM modules should be adapted to VH, telehealth, and e-manual delivery to create a 5- or 10-week program, both of which have been shown to be efficacious in reducing distress with human delivery [72,73]. A pilot study should be conducted to compare the feasibility and acceptability of the technologies when delivering a home-based 5- or 10-week CBSM intervention followed by a fully powered RCT to evaluate efficacy. Potential attention-matched control conditions such as health education could also be compared. A trial conducted over a longer period (eg, 5 or 10 weeks) should compare intervention engagement between the conditions. This study was limited in its ability to evaluate engagement in 1 session, and a VH with social engagement might be associated with greater use in a longer-term, home-based trial. In addition, further research is needed to evaluate these technologies while providing other types of psychological interventions in broader populations. This includes in diverse cultural contexts, age groups, and clinical populations. Different technologies may provide a better fit in certain populations or use cases. Indeed, the qualitative results of this study revealed the unique strengths of each delivery method. Additional research is needed to explore the intervention contexts and populations in which these technologies are best suited.

Further research is needed to better understand the factors that contribute to perceptions of rapport and trust with VH. Previous literature has identified factors that contribute to trust with social robots (eg, robot personality, reliability, appearance, behavior, error mitigation, and personalization) [74,75] and conversational agents more broadly [43,62,66]. Similar factors may be relevant to VH, and research could specifically explore the effects of multimodal sensing of user states and expressions of empathy on intervention engagement.

Other areas for future research to investigate is which intervention technology people prefer when given a choice, and how evaluations of the technologies differ in a within-subjects design where participants are exposed to all 3 conditions. Research could also examine the effect of choice on the acceptability of VH. Research has shown that treatment satisfaction, completion, and clinical effects are greater when users receive psychological treatment that they have chosen [76], and having a choice of treatment may reflect how these technologies would be used in the real world. Research should also explore how choosing the design of one’s VH affects acceptability. Studies have shown that the demographic and appearance characteristics of VH can affect competence perceptions and preferences, depending on the degree of similarity to the user [77].

### Conclusions

On the basis of the overall data, a VH, teletherapy, and a self-guided e-manual were feasible and acceptable ways to provide 1 session of a stress management intervention to a community sample of distressed adult women. Unique strengths and limitations were identified for each technology, and further trials are needed to identify the populations and use contexts that each technology is best suited to. Future research should develop and test a longer 5- or 10-week CBSM intervention with these delivery methods and use a similar methodology to conduct an RCT that compares the feasibility, acceptability, and effectiveness of the technologies when delivering a longer, home-based intervention. All 3 technologies have the potential to expand the reach of stress management interventions; however, more trials are required.

## Appendix

Multimedia Appendix 1 An overview of the research procedure.

Multimedia Appendix 2 Definitions for themes in response to the qualitative questions.

Multimedia Appendix 3 Themes, subthemes, and representative quotes describing strengths and limitations of the homework exercises.

## Abbreviations

CBSM: cognitive behavioral stress management
CONSORT: Consolidated Standards of Reporting Trials
E-CBSM: e-manual cognitive behavioral stress management
RCT: randomized controlled trial
T-CBSM: teletherapy cognitive behavioral stress management
VAS: Visual Analog Scale
VH: virtual human
VH-CBSM: virtual human cognitive behavioral stress management
WAI: Working Alliance Inventory

## Data Availability

Under the ethics agreement of this research, participants were informed that their data would remain confidential to the research team. The participants did not consent for their data to be made publicly accessible. The corresponding author can be contacted for any queries.
