# Supplementary figures and images for: Comparing the Feasibility and Acceptability of a Virtual Human, Teletherapy, and an e-Manual in Delivering a Stress Management Intervention to Distressed Adult Women: Pilot Study
Source: JMIR Form Res. 2023 Feb 9;7:e42390. doi: 10.2196/42390 (PMC9951078; doi:10.2196/42390)

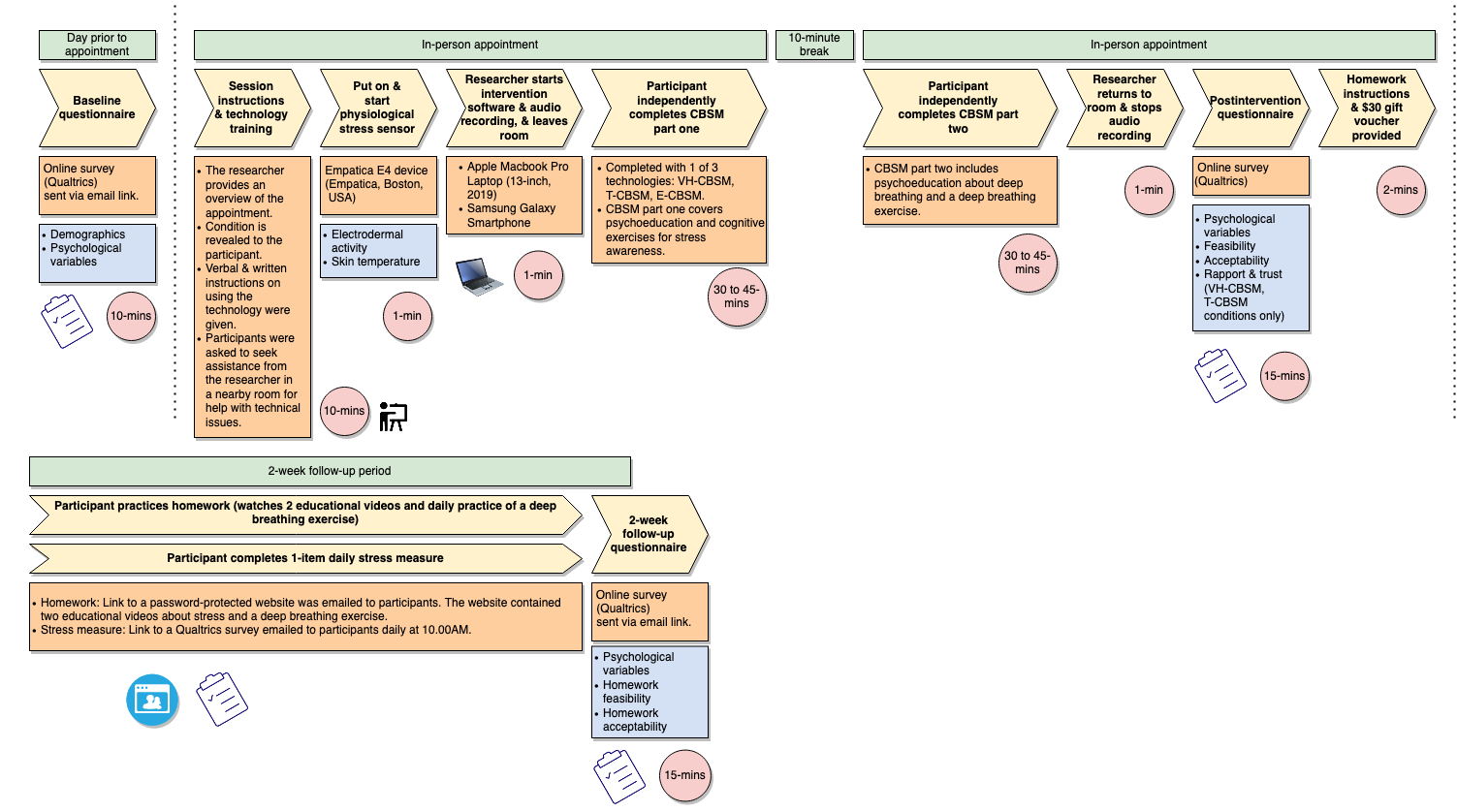

Supplement: Multimedia Appendix 1 [file formative_v7i1e42390_app1.png]
