# Supplementary material for: Comparing the Feasibility and Acceptability of a Virtual Human, Teletherapy, and an e-Manual in Delivering a Stress Management Intervention to Distressed Adult Women: Pilot Study
Source: JMIR Form Res. 2023 Feb 9;7:e42390. doi: 10.2196/42390 (PMC9951078; doi:10.2196/42390)
Supplement: Multimedia Appendix 2 [file formative_v7i1e42390_app2.docx]

## Multimedia Appendix 2

## Definitions for themes in response to the qualitative questions:

| *“What did you like about the delivery of your therapy session?”* | |
| --- | --- |
| Theme | Definition |
| Therapist qualities | Personal characteristics of the human therapist. |
| Teletherapy delivery | Aspects of receiving therapy through video call. |
| AI delivery | Aspects of receiving therapy from an artificially intelligent computer agent. |
| Self-delivery | Aspects of self-guided therapy. |
| User experience | Components of the technology interface or therapy experience. |
| Therapy content | The information or exercises included in the therapy, or the manner in which these were delivered. |
| Environment | Aspects of the clinical room in which the therapy session took place. |
| *“How do you think the delivery of the therapy session could be improved?”* | |
| Theme | Definition |
| Delivery style | The manner in which the therapy information and exercises were delivered. |
| Therapy content | Aspects that could be added to or removed from the therapy information or exercises. |
| Environment | Aspects of the clinical room in which the therapy session took place. |
| Digital human design | Aspects of the Digital Human’s appearance, interface, or speech. |
| No improvement | No changes were requested. |
| *“What did you like about the homework exercises?”* | |
| Theme | Definition |
| Breathing exercises | Benefits of the deep breathing exercise, including its psychological effects and ease of use. |
| Videos | Aspects of the videos, pertaining to their content and length. |
| Daily stress assessment | Benefits of the daily stress assessment, including its psychological effects. |
| Experience | Positive aspects of engaging in the homework exercise, including psychological benefits and ease of use. |
| *“How do you think the homework exercises could be improved?”* | |
| Theme | Definition |
| Videos | Aspects of the homework videos pertaining to their content, age, and timing of delivery. |
| Content | Therapeutic exercises that could be added to the homework. |
| Trial methods | Changes to the trial methods which would improve the delivery and assessment of the homework exercises. |
| No improvement | No changes were requested. |
