# Supplementary material for: Comparing the Feasibility and Acceptability of a Virtual Human, Teletherapy, and an e-Manual in Delivering a Stress Management Intervention to Distressed Adult Women: Pilot Study
Source: JMIR Form Res. 2023 Feb 9;7:e42390. doi: 10.2196/42390 (PMC9951078; doi:10.2196/42390)
Supplement: Multimedia Appendix 3 [file formative_v7i1e42390_app3.docx]

## Multimedia Appendix 3

| Themes | Subthemes | Representative quotes |
| --- | --- | --- |
| *Teletherapy* | | |
| Therapist qualities | Calm | *“Nice and calm talking through the intervention.” [038]* |
|  | Attentive | *“The therapist was attentive.” [037]* |
|  | Calming voice | *“Calming voice.” [021]* |
|  | Comfortable | *“Made you feel comfortable.” [030]* |
| Teletherapy delivery | Available remotely | *“Everything. The fact that it was on Zoom makes me think that I could do it anywhere (i.e., at work) and would not waste time changing places.” [009]* |
|  | No technical difficulties | *“No difficulties while using Zoom.” [036]* |
| User experience | Delivered by a person | *“Nice to have a person guide you through the process.” [024]* |
|  | Ease of use | *“It was easy.” [017]* |
| Therapy content | Not too personal | *“Not delving too deep.” [026]* |
|  | Practices during session | *“Doing practices during the session.” [002]* |
| *Virtual Human* | | |
| AI delivery | Non-judgmental | *“Non-judgmental since there was no human.” [018]* |
|  | No social pressure | *“It is interactive but more relaxing than facing a real person.” [032]* |
|  | Humanlike | *“She was quite humanlike, impressive for a virtual person, and most of the intonation was good.” [031]* |
|  | Hold eye gaze | *“When responding to an actual person, you wouldn’t actually hold their gaze as much as you do with an AI. I liked that.” [035]* |
|  | Factual | *“The unemotional delivery of the AI. Everything was factual.” [014]* |
|  | Good responses | *“I like Sam, she responded well to verbal answers.” [029]* |
|  | Sam | *“The Digital Human therapist.” [043]* |
| User experience | Ease of understanding | *“It was easy to understand.” [008]* |
|  | Ease of use | *“Ease of use.” [001]* |
|  | Speech speed | *“Speech speed was good.” [001]* |
|  | Supporting illustrations | *“I liked the visual aspects of the session as sometimes I found the detailed information a bit much to take in, coming from a non-medical background.” [014]* |
|  | Interaction options | *“That you can also type answers/click on responses.” [019]* |
| Therapy content | Not too personal | *“It was not too personal (i.e., did not have to provide any details about my life.” [005]* |
|  | Rich content | *“The content was rich but not too overwhelming.” [032]* |
| *e-Manual* | | |
| Self-delivery | Privacy | *“Privacy. Time to reflect and let the information sink in.” [003]* |
|  | Self-paced | *“Could be done at your own pace.” [028]* |
|  | No social pressure | *“I liked that I could take my time and think about my answers without someone in the room making me feel pressured.” [034]* |
| User experience | User friendly and engaging | *“The electronic parts were user friendly and engaging. The audio was good.” [027]* |
|  | Illustrations | *“The pictures were a nice touch!” [006]* |
|  | Forms | *“I liked the parts that I could fill out my information in.” [006]* |
| Therapy content | Clear instructions | *“Instructions are well set out.” [007]* |
|  | Professional | *“Professional and friendly.” [004]* |
|  | Good explanations | *“It’s well explained before completing the questions. This makes sure we understand the material so that we can provide correct responses.” [020]* |
| Environment | Quiet room | *“A quiet room, clear simple instructions.” [033]* |

Table 3. Themes, subthemes, and representative quotes describing what participants liked about the delivery of the intervention.

| Themes | Subthemes | Representative quotes |
| --- | --- | --- |
| *Teletherapy* | | |
| Delivery style | Simplify language | *“It would be helpful to explain things in more everyday terms. Some explanations were quite technical, e.g., the information I had to read. Not sure all the details added to my understanding of stress.” [024]* |
|  | Tailor suggestions | *“Perhaps asking more about the different stress management techniques already being used so could deepen and improve techniques to do this.” [038]* |
|  | Avoid reading script | *“…something a little unnerving about a person reading from a script.” [010]* |
|  | In-person delivery | *“In person? Otherwise no improvements.” [017]* |
| Therapy content | Shorter session | *“Make the session a bit shorter.” [002]* |
|  | Less reading | *“By cutting off the reading part…” [002]* |
|  | Option to lie down during deep breathing | *“Lying down for relaxation/ deep breathing.” [026]* |
| Environmental | Background music | *“Sometimes have music maybe.” [025]* |
|  | Desktop computer | *“Bigger screen and a proper keyboard (ergonomics).” [013]* |
| No improvement | N/A | *“Nothing in particular. I enjoyed a lot the way the therapy session happened.” [009]* |
| *Virtual Human* | | |
| Delivery style | Simplify language | *“Communication and language used could possibly be simpler for the average person to digest.” [014]* |
| Therapy content | Shorter session | *“Long session.” [035]* |
|  | Less information on the brain | *“Much less on how the brain works.” [018]* |
|  | Stress video – more accessible language | *“There were parts throughout the stress response explanation video where I thought the language could have been more direct and simpler to connect to those such as myself with no medical background.” [014]* |
|  | Stress video – more engaging delivery | *“I can’t really remember the [video] content as at the time I found it very disengaging.” [018]* |
| Digital human design | More tailored responses | *“Maybe more tailored information depending on the responses to multiple-choice questions.” [005]* |
|  | Slower speech speed during explanations | *“Modulating the speed better. She speaks quite fast which is fine for short or simple instructions, but gets a bit much for the more detailed explanations.” [031]* |
|  | Improve sync of lips to speech | *“Voice and face/lips movements of the Digital Human synchronized.” [018]* |
|  | Ability to select multiple response options | *“Some of the questions are multiple-choice, but I only got to click on one option before the screen moved on.” [032]* |
| Environmental | Warmer room | *“…it got cold as I am used to moving a lot.” [035]* |
|  | More comfortable chair | *“More comfortable chair…” [035]* |
| No improvement | N/A | *“I don’t think it needs improvement.” [001]* |
| *e-Manual* | | |
| Delivery style | Simplify language | *“Maybe simpler language?” [003]* |
|  | More interactivity | *“I would prefer something more interactive, perhaps with some reading, some audio, some questionnaires, etc.” [006]* |
|  | Have a facilitator present | *“Having no one around to prompt me or help me think through the different stressor situations and how I responded could mean that I was missing something. Being alone for the delivery could make people feel less accountable- maybe they won’t do it properly or rush through it.” [034]* |
|  | In-person deep breathing exercise | *“Personally, I would like to do the breathing exercises with a human, rather than guiding by the computer.” [020]* |
| Therapy content | Less reading | *“Less reading at the beginning.” [028]* |
|  | More visual components | *“I think rather than significant chunks of reading, a more visual powerpoint may make things easier to understand.” [041]* |
| Environmental | More comfortable chair | *“The chair was not particularly comfortable.” [033]* |
|  | Desktop computer | *“Not on a laptop.” [016]* |
|  | Dim lights | *“Dimming the lights might.” [007]* |
|  | More comfortable environment | *“A more comfortable/ less clinical setting.” [028]* |
| No improvement | N/A | *“It was fine.” [004]* |

Table 4. Themes, subthemes, and representative quotes describing what participants thought could be improved about the delivery of the intervention.

| Themes | Subthemes | Representative quotes |
| --- | --- | --- |
| Breathing exercises | A strategy to cope with stress | *“In times when I felt more stressed I would do the homework exercise. I think it helps.” [007]* |
|  | Helpful to mood | *“Good to have to set aside time to do something good for me and helpful to my mood.” [042]* |
|  | Able to do across situations | *“They were simply explained and flexible to be able to do in a variety of situations.” [028]* |
|  | Able to do in one’s own time | *“I could do them in my own time and preferred location.” [029]* |
|  | Simple | *“They are relatively simple.” [033]* |
| Videos | Reinforced therapy content | *“It’s simple and a quick reminder of what I have learned at the session.” [032]* |
|  | Audio to support deep breathing practice | *“I particularly liked the breathing video as for some reason when someone is talking to me and telling me how to breathe it seems to work better for me.” [034]* |
|  | Short length | *“The videos were short.” [008]* |
| Daily stress assessment | N/A | *“I liked the daily reminder to assess my level of stress, it prompted me to actively work on reducing the stress where I could, breathe more deeply, take a break from the computer and enjoy nature too.” [013]* |
| Experience | Increased confidence | *“I felt more confident in doing the deep breathing by myself.” [009]* |
|  | Easy to do | *“Easy to access. Watching videos is an easy task.” [012]* |
|  | A reminder to practice | *“I enjoy mindfulness regularly anyway- was a good reminder to do it almost each day.” [003]* |

Table 5. Themes, subthemes, and representative quotes describing what participants liked about the homework exercises.

| Themes | Subthemes | Representative quotes |
| --- | --- | --- |
| Videos | Update videos | *“Felt like the videos were really old school – in terms of the quality.” [008]* |
|  | Remove repeated information | *“The videos seemed a bit repetitive which I disengaged from. There was a lot of information that was the same as what had already been covered – I understand the need to reiterate the learning, but rather than serving as a reminder it made me want to skip through.” [006]* |
|  | Tailor to the local cultural context | *“Very US centric – a more culturally appropriate video would have been good.” [024]* |
|  | Shorten | *“Too long- adds to the stress of fitting them in.” [035]* |
|  | Time delivery | *“I would suggest to ask people to watch the video that teaches how to breathe again after a week. It might be good to remember how to do the breathing and to create a habit of doing the deep breathing daily.” [009]* |
| Content | Teach a new stress management technique | *“Introduce a different technique perhaps?” [016]* |
|  | Teach how to cope with negative thoughts | *“I think there could have been some suggestions on how to deal with negative thoughts.” [038]* |
| Trial methods | Send reminders | *“It would be great to be sent a reminder about the videos along with the daily stress level checks. I may have watched them more actively if I were prompted to (because the link was readily available).” [126]* |
|  | Ask for video feedback immediately after watching | *“Questions regarding the exercises could be asked after the participant has finished viewing them (whenever that is).” [016]* |
| No improvement | N/A | *“Can’t see any improvements – it was easy to do.” [017]* |

Table 6. Themes, subthemes, and representative quotes describing how participants felt the homework exercises could be improved.
